# A method for detailed analysis of the structure of mast cell secretory granules by negative contrast imaging

**DOI:** 10.1038/srep23369

**Published:** 2016-03-21

**Authors:** Shotaro Tanaka, Yuichi Takakuwa

**Affiliations:** 1 Department of Biochemistry, School of Medicine, Tokyo Women’s Medical University, Kawada 8-1, Shinjuku, Tokyo 162-8666, Japan

## Abstract

Secretory granules (SGs) in mast cells contain various molecules that elicit allergy symptoms and are generally considered therapeutic targets. However, the biogenesis, maintenance, regulation, and recycling of these granules remain controversial, mainly due to the lack of suitable live-cell imaging methods. In this study, we applied negative contrast imaging with soluble green fluorescent protein (GFP) expressed in the cytoplasm as a method to validate structural information of mast cell SGs. We evaluated the accuracy of the method in detail, and we demonstrated that it can be used for quantitative analysis. Using this technique, secretory granules, the nucleus, mitochondria, and the cell body were visualized in individual RBL-2H3 mast cells without any influence. When combined with conventional multicolor fluorescence imaging, visualization of SG-associated proteins and SG–SG fusion was achieved. Moreover, 3D images were constructed based on this method, and detailed information on the number, size, and shape of individual SGs was obtained. We found that cell volume was correlated with SG number. In summary, the technique provides valuable and unique data, and will therefore advance SG research.

Mast cells maintain a large number (~1,000) of secretory granules (SGs) in the cytoplasm^1^. These granules are tightly regulated and contain various molecules that elicit inflammation, including histamine, serotonin, and several proteases^2^,^3^. Formation of a complex between immunoglobulin E and its high-affinity receptor FcεRI triggers multiple tyrosine kinase cascades^4^, whereupon SGs anchored beneath the plasma membrane immediately fuse to it^5^. Upon fusion, the granules extrude cargo into the extracellular space, an event followed by onset of allergy symptoms. Several palliative strategies, such as inhibiting the histamine H1 receptor^6^, have been developed. However, directly inhibiting SG generation, maintenance, regulation, or recycling is likely to be more effective. Therefore, SG biogenesis must be urgently investigated.

In the past decades, SGs have been characterized, mainly by electron microscopy, at every stage of maturation. The generally accepted model of SG biogenesis was proposed by Hammel *et al*.^7^,^8^. In this model, numerous uniformly sized pre-granules bud from the Golgi apparatus, aggregate, and fuse to generate immature granules. These immature granules eventually mature^7^, and are then categorized into three types based on cargo^7^,^8^,^9^. In addition, transport vesicles from the late endosome may integrate with immature granules^2^,^9^. These studies suggest, as noted, that a single mast cell contains a large number of SGs of various types and in various stages of development^2^,^10^.

In addition, SG development has been studied using immunofluorescence or gene knockdown and knockout of associated proteins. For example, Rab5A, a small GTPase and marker of the early endosome, has been shown to transiently bind SGs that bud from the Golgi apparatus^11^, and is thus considered a marker of pre-granules or immature granules. Rab3D^12^ and secretogranin III^13^ have been demonstrated to be involved in SG biogenesis. Rab27b^14^, vesicle-associated membrane protein-8 (VAMP-8)^10^, and syntaxin-3^15^ are required for degranulation, indicating that they interact with mature SG. Synaptotagmin III targets mature SG to the endocytic recycling complex^16^. SGs also contain lysosomal proteins and markers such as CD63^17^ and β-hexosaminidase^18^, and are therefore called lysosomal granules^19^.

The next frontier is to characterize the structure and spatiotemporal organization of SGs as they mature. Therefore, it is necessary to observe and trace SGs in live cells and in real time. Unfortunately, live-cell imaging using conventional techniques is problematic. For instance, these techniques require marker proteins such as CD63 or neuropeptide Y^19^,^20^ fused to fluorescent proteins. However, the granules cannot be traced throughout their lifetime if the interaction with the marker protein is temporal. In addition, there is no known universal marker for all SG classes^2^ and this technique cannot be used to investigate SGs with no known markers, such as those that have atypical or unknown origin. Finally, overexpression of SG-binding proteins may perturb SG structure and activity. Thus, a new approach is required to directly visualize native SGs in live cells.

One potent method to resolve the problem is negative contrast imaging (NCI) with fluorescent protein expressed in the cytoplasm^21^,^22^,^23^,^24^. In the cells, organelles including SGs are separated from the cytoplasm by a lipid bilayer. As these organelles exclude soluble fluorescent protein expressed in the cytoplasm, negatively stained organelles can be visualized by evanescent-field micrography^22^, two-photon microscopy^23^, and confocal microscopy^21^,^23^,^24^. NCI does not require the use of organelle markers; however, the use of fluorescent probes does not affect NCI^21^,^22^,^23^,^24^. NCI is suitable for reconstructing lateral view and 3D images^23^,^24^ because this technique provides clear contrast.

In this report, we applied NCI for detailed analysis of mast cell SGs. Using NCI, we observed native SGs in live mast cells without using markers. We confirmed the accuracy and fidelity of the method by utilizing microbeads *in vitro* and in live mast cells. Furthermore, we combined NCI with conventional fluorescence imaging to visualize interactions between SGs and associated proteins, and to observe SG–SG fusion between marked and unmarked granules. In addition, we reconstructed 3D images of individual mast cells, and found a correlation between cell volume and SG number, and between SG volume and SG number. Thus, this method enables real-time observation of SG development, as well as the analysis of associated cell structures.

## Results

### Visualization of all SGs by NCI

**Figure 1:**
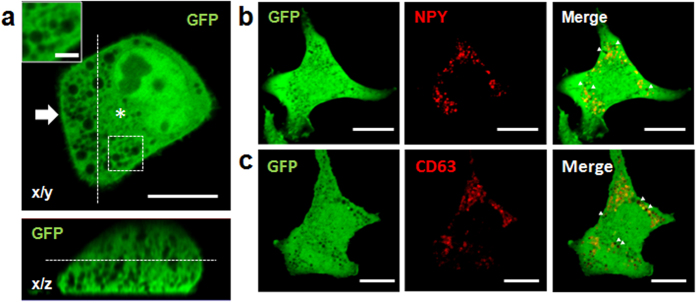
Negative contrast imaging of secretory granules in live mast cells. (**a**) Horizontal (top) and lateral (bottom) views of a live RBL-2H3 cell expressing GFP. Inset is a high-magnification view of the boxed region, and dashed lines indicate cross sections. Scale bars are 10 μm in the main image and 2 μm in inset. (**b,c**) Confocal image of a live RBL-2H3 cell co-expressing GFP and NPY–mCherry (**b**) or GFP and CD63–mCherry (**c**). Arrowheads highlight negatively stained organelles unmarked with mCherry. Scale bar, 10 μm.

We first attempted to visualize organelles in RBL-2H3 rat mast cells transiently expressing GFP in the cytoplasm. Many unstained organelles with 0.5–1.0-μm diameter were distinctly outlined against a fluorescent field (Fig. 1a). Small, obscure o utlines were also observed in the perinuclear region (Fig. 1a, asterisk). These outlines were surrounded by large, distinct, spherical shapes. The generic SG markers neuropeptide Y (NPY) and CD63 mostly localized to areas of high negative contrast (Fig. 1b,c), indicating that these organelles are SGs. This result suggested that NCI can be combined with conventional imaging techniques based on fluorescently labeled SG markers. Notably, we also observed organelles with similar shape and size as SGs, but that were not stained with the SG markers (Fig. 1b,c arrowhead).

NCI also outlined the cell body, as well as nuclear structures like nucleoli and the nuclear membrane (Fig. 1a). We observed mitochondria as small, tubular-shaped organelles that were negatively stained with GFP, but that were stained with MitoTracker^®^, a mitochondria-specific dye (Supplementary Fig. S1).

Nevertheless, we observed varying degrees of negative staining in the nucleus that presumably highlight the nucleoli, Cajal bodies, and other nuclear components^25^. We hypothesize that such negative contrasts are generated because highly condensed structures such as these prevent GFP from freely diffusing into the core. Indeed, the ability to observe these features is a notable advantage of NCI, and may prove useful in investigating the cell cycle. However, more experiments are necessary to validate this interpretation.

Previous studies have utilized NCI for visualization of secretory vesicles and SGs in other cell lines^21^,^22^ and in cells in live organs^23^,^24^. To confirm whether SGs in other cell lines can also be negatively stained using the method presented here, we tested NCI in G361^26^ skin cells. These cells contained many negatively stained organelles as well, although the pattern of staining was different from that in mast cells. Skin cells contained numerous small (0.5–0.8 μm) organelles (Supplementary Fig. S2). These organelles were spherical and individually separated when viewed from the x/z axis (Supplementary Fig. S2, arrowheads), whereas SGs in mast cells were stretched along the z axis, and were either single elongated structures, or formed a cluster of structures strung together (Fig. 1a, lower panel).

Taken together, our results indicated that NCI can be used in various cell lines to visualize large organelles such as SGs while preserving native structure and function.

### Accuracy and fidelity of NCI

We next evaluated the accuracy and fidelity of NCI *in vitro* and in cells (Supplementary Fig. S3). First, uniformly sized polystyrene beads of 0.5, 0.75, and 1.0 μm diameter were labeled with AF555 red fluorescent dye and suspended in a solution of GFP. Next, we imaged the slurry using NCI, and reconstructed 3D images. The beads had no detectable autofluorescence in the range of fluorescence observed, and the excitation and emission laser beams had good penetration. In deconvoluted images, negatively stained volumes were found to be nearly spherical (Supplementary Fig. S3a, and inset in Supplementary Fig. S3c) with a diameter (Supplementary Fig. S3b) and volume (Supplementary Fig. S3c) nearly equal to the corresponding theoretical values of a 1.0-μm sphere (98.7 ± 5.0% as diameter, n = 12). However, results were inaccurate for beads >1.0 μm or <0.5 μm in diameter. The contours of these beads were obscured because fluorescence did not penetrate as effectively around large beads, while it diffracted around small beads. Similar results were obtained when the beads were delivered by electroporation into live mast cells expressing GFP (Supplementary Fig. S3d–f). These results demonstrated that NCI precisely captures the structure of intracellular organelles, at least those 0.5–1.0 μm in diameter, which is typical of mast cell SGs.

### SG shape, size, and volume

**Figure 2:**
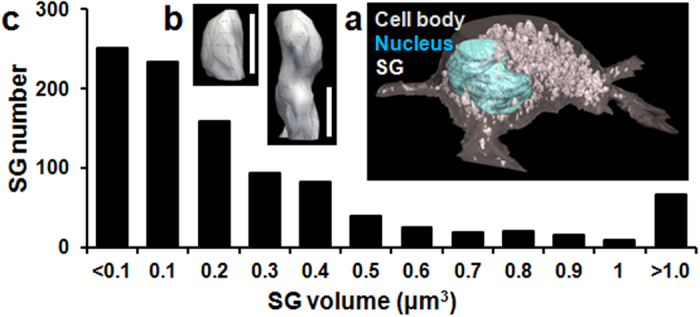
3D reconstruction of a mast cell. (**a**) A 3D image of the same cell in Fig. 1a. SGs are illustrated in white, while the nucleus and cell body (3,338 μm^3^) are depicted in cyan and grey, respectively. (**b**) 3D images of representative SGs with volume 1.32 μm^3^ (right) and 0.41 μm^3^ (left). Scale bar, 1.0 μm. (**c**) Distribution of SG volume and SG number (n = 1,014) in the same cell.

The negative contrast in NCI provides clear and distinct contours (Fig. 1). Therefore, we used NCI to reconstruct a 3D image of a cell and to determine the shape, size, and volume of SGs. To distinguish individual SGs, we laminated the contours of organelles and the cell body as abstracted from serial deconvoluted NCI images. To achieve lamination, we typically acquired serial confocal images at 0.2-μm intervals (about 70 images per cell). From the 3D reconstruction (Fig. 2a), we obtained the number and size of all detectable SGs, as well as the volume of the cell body. As has been noted, many 3D images show SGs to be composed of multiple spherical SGs strung together (Fig. 2b right panel, representative). Nevertheless, the distribution of the volume and number of SGs in a cell (Fig. 2c) was consistent with electron microscopic data^7^.

**Figure 3:**
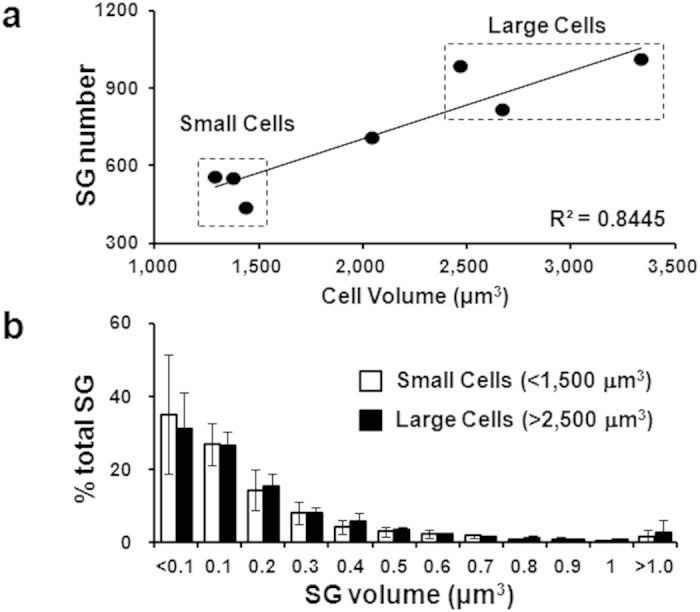
Correlation of SG number with cell volume. (**a**) Distribution of cell volume and SG number (n = 7) in GFP-expressing RBL-2H3 cells. (**b**) Distribution of SG volume and number in small (open bars) and large (filled bars) cells marked in (**a**). Data are expressed as the mean + SD.

Further, we reconstructed 3D images of several cells of various sizes, and found the number of SGs to positively correlate with cell volume (Fig. 3a). Indeed, the relationship between cell volume and SG number in small and large cells is similar to that between mother and daughter cell(s), as measured in time-lapse observation of cells undergoing mitosis (data not shown). On the other hand, the size distribution of SGs was comparable between small (<1,500 μm^3^) and large (>2,500 μm^3^) cells (Fig. 3b). These results indicated that SGs increase in abundance with cell size, but are under continuous control to maintain size distribution.

### NCI visualizes SGs without markers

**Figure 4:**
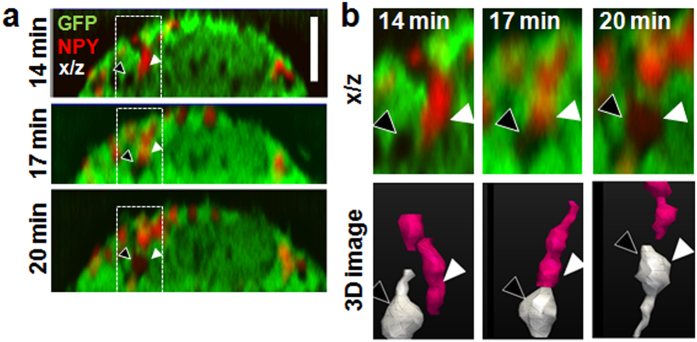
Visualization of SG-SG fusion in PMA-activated RBL-2H3 cells. (**a**) Lateral view of a representative cell expressing GFP and NPY–mCherry. Images of the same cell were acquired 14 (top), 17 (middle), and 20 (bottom) min after PMA activation. Arrowheads highlight SG–SG fusion events. (**b**) High-magnification view (top) and 3D image (bottom) of the boxed region in (**a**), which shows SGs with (magenta) or without (white) NPY. Dashed lines are cross sections. Scale bar, 5 μm.

We used NCI in combination with conventional multicolor fluorescence imaging to visualize SG–SG fusion in live mast cells (Fig. 4). We added PMA to induce SG–SG fusion and pinocytosis in mast cells co-expressing GFP and mCherry-labeled neuropeptide Y. PMA induced membrane ruffling (compare Fig. 1a, lower panel with Fig. 4a, upper panel), and obvious SG–SG fusion within several minutes, as has been reported^27^. Figure 4 shows a representative image of fusion between SGs with and without the neuropeptide. In this image, taken approximately 14 min after PMA addition, an unstained SG (black arrowhead) coalesced with a stained granule (white arrowhead) and fused with it at around 20 min. The fluorescence per unit volume in the resulting SG decreased as a result (Fig. 4a,b 20 min, arrowhead). Notably, almost all SG–SG fusion events seemed to occur along the same axial direction as in Fig. 4.

## Discussion

It is reasonable to assume that directly inhibiting SG biogenesis will reduce allergy symptoms. Unfortunately, the detailed molecular mechanism driving this process remains unknown, mostly because of a lack of suitable imaging methods. To address this issue, we applied NCI to live mast cells, and we were able to directly visualize all SGs with sufficient contrast to reconstruct 3D images. We anticipate that this method will facilitate molecular studies of SG biogenesis in mast cells.

We demonstrated that NCI is a powerful method to directly visualize all SGs in their native state in live mast cells and in real time. Theoretically, NCI will negatively stain all structures that exclude GFP expressed in the cytoplasm, including most organelles separated by a lipid bilayer. Therefore, the method can be used to visualize all SGs in various developmental stages without using markers, although it remains limited by the resolution of the microscope. In addition, freely diffusing GFP, or any other fluorescent protein, is generally regarded to have little interaction with other molecules or organelles, and therefore, should not interfere with SG activity. We also showed, using microbeads similar in size to mature SGs, that NCI captures precise structural information (Supplementary Fig. S3). Collectively, these observations strongly indicate that negatively stained structures in the cytoplasm are in the native state. Nevertheless, NCI is an indirect imaging method. However, the technique provides real-time information with or without qualitative or structural changes in the organelle, at least until GFP somehow reaches its lumen. Therefore, we believe that NCI provides high-quality structural information that is challenging to obtain with direct methods, and thus, is more suitable for investigating SG biogenesis in mast cells.

By imaging polystyrene beads with diameter 0.5–1.0 μm *in vitro* (Supplementary Fig. S3b, c) and in cells (Supplementary Fig. S3e,f), NCI was demonstrated to capture accurate structural information. This is likely because of the fact that the method outlines objects more sharply than fluorescently labeled SG markers (Fig. 1c, Supplementary Fig. S3a), which positively stain vesicles by accumulating on the cytoplasmic leaf of the SG membrane. However, the concentration of fluorescence also enhances scattering and diffraction, thereby reducing contrast and blurring outlines, especially in cells that contain up to 1,000 SGs^1^. Indeed, this issue has severely limited SG research. On the other hand, diffraction and scattering are less of an issue in NCI, where organelles are negatively stained, and fluorescence does not accumulate on the SG surface. Instead, diffraction facilitates the reconstruction of relatively small structures in 3D images (Supplementary Fig. S3a–f), providing another opportunity to precisely trace the boundary of individual SGs. Informative results are also obtained when NCI is combined with fluorescently labeled cargo (Fig. 1b and Fig. 4). For example, expressing red fluorescent protein-fused NPY in live mast cells allowed real-time visualization and quantitative assessment of degranulation^20^, which is difficult to precisely detect by using NCI alone; however, not all SGs accumulate NPY in the lumen (Fig. 1b). We are now studying degranulation of live mast cells expressing both soluble GFP in the cytoplasm and NPY–mCherry.

We reconstructed accurate 3D images of whole cells from serial NCI images (Fig. 2a) and obtained structural information on all recognizable SGs, including diameter, volume, shape, number, and location in the cell. The size distribution of SGs (Fig. 2c) was obtained for the first time in live mast cells, and these data may prove useful to classify SGs by size or shape (Fig. 2b,c). We speculate that integration of immunofluorescence data with NCI structural data may provide a detailed taxonomy of SGs in live cells and help illuminate the SG life cycle. Furthermore, visualization of structural changes in SGs may help characterize the effects of regulatory molecules^20^ and drugs. Several drugs that reduce degranulation (or stabilize mast cells) through unknown mechanisms^28^ may indeed affect SG generation or SG–SG fusion, events that can be observed by NCI.

Interestingly, we observed that typical SGs have extended structures along the z axis (Fig. 1a, lower panel and Fig. 2a,b), and that fusion among SGs frequently occurs along the same axis (Fig. 4). To minimize z-axis artifacts in orthogonal and 3D images obtained by serial confocal imaging, we deconvoluted images, evaluated accuracy using microbeads (Supplementary Fig. S3), and imaged G361 cells (Supplementary Fig. S2), which is frequently used in SG research. Based on these experiments, we found that orthogonal views and 3D images obtained by NCI are accurate, although complete elimination of artifacts remains challenging. These data strongly suggest that SGs in mast cells are somewhat cylindrical, and fuse along the vertical axis (Fig. 4), indicating polarity in both structure and fusion. Nevertheless, NCI remains limited by the resolution of the microscope, especially in the z direction. Consequently, we could not definitively establish whether cylindrical SGs (Fig. 1) are individual structures or consist of several structures tightly clustered.

Based on 3D reconstructions, we found a positive correlation between cell volume and SG number, and between SG volume and SG number (Fig. 3). These results are consistent with electron microscopic data^7^. We also observed that even though the volume of the cell changes over the cell cycle (data not shown), the distribution of SG volume and number was tightly preserved throughout (Fig. 3b), suggesting stringent regulation of SG biogenesis and maintenance. This result is important, because proliferation of mast cells in peripheral tissues^29^ is an issue of interest in allergy treatment: in patients with mastocytosis, the increased density of mast cells in peripheral tissues is attributed to unregulated proliferation^30^. In light of these results, we believe that NCI will prove useful in investigating the cell cycle because it minimally interferes with SG structure and function, and allows simultaneous imaging of the SGs, cell body, and nucleus.

One of the most obvious advantages of NCI is the ability to visualize marker-negative SGs. In Fig. 4, we successfully observed for the first time SG–SG fusion between SGs with and without markers. The data indicate that SG–SG fusion typically occurs along just one axial direction (Fig. 4). Therefore, imaging experiments should be set up accordingly. For example, it is possible to capture fusion events involving neuronal secretory vesicles in a single scan because these vesicles are approximately 50 nm in diameter^1^, while a standard confocal microscope has focal spot between 0.5 and 1.0 μm. On the other hand, serial tomographic scans are required to observe similar events in mast cells, in which SGs are approximately 0.3 to 1.0 μm wide^1^. 3D imaging is crucial in this instance. NCI is suitable for such visualization experiments, because it provides high-contrast images, enables simultaneous imaging of the cell body and the nucleus, and does not suffer from photobleaching as a result of abundant GFP expression.

A number of practical issues remain. First is the lack of molecular evidence to show that vesicles unstained by SG markers are, indeed, SGs. This is significant as molecular-based classification of SGs in live cells is emerging as key to understanding biogenesis. Therefore, we are currently investigating ways to combine NCI with a panel of fluorescently labeled SG markers as reported previously^20^, including SG-associated proteins (v-SNARE, Rab), cargoes (SG-specific protease, cytokine, peptidoglycan), and lipid-binding proteins (phosphatidylserine-binding protein). We anticipate that this approach will provide a detailed taxonomy of SGs that corresponds to electron microscopic data^7^. Of note, careful consideration is needed when choosing fluorescent protein(s) as additional beacon to avoid interference with NCI. Second is the deterioration of image quality due to movement, as acquisition of a single 3D image presently requires 1–2 min. In live cells, organelles such as trafficked vesicles are easily relocated within a few seconds. Therefore, a serial scan acquired over several minutes will be substantially degraded. Such effects may be minimized by increasing the acquisition rate, such as, for example, in spinning-disk confocal microscopy. Alternatively, cells may have to be fixed, although preliminary experiments suggest that fixing with paraformaldehyde distorts SG morphology (data not shown). Last is the difficulty of image processing and analysis. SGs are densely packed in confocal images (Fig. 1a), and are challenging to separate programmatically. Therefore, several hours are currently required to reconstruct a 3D image from scans acquired in a few minutes. We are now exploring ways to automate and accelerate this process.

In summary, we describe in this paper NCI as an imaging method to visualize all secretory granules in live mast cells without using fluorescently labeled marker proteins. NCI enables 3D imaging of SGs and other cell bodies, and captures fusion events, as well as structural information such as organelle volume, size, and number. The technique complements conventional imaging very well; therefore, it will be a valuable tool in SG research.

## Methods

### Plasmid construction

The GFP coding sequence was amplified by PCR from pEGFP-N1 (Clontech Laboratories Inc., Mountain View, CA, USA) and inserted between the *Hin*dIII and *Eco*RI restriction sites in pcDNA4/myc-HisB (Invitrogen/Life Technologies, Grand Island, NY, USA). The NPY–mCherry fusion was constructed as previously described^31^. Briefly, the NPY coding sequence (amino acids 1–97) was amplified by PCR from human bone marrow Quick-Clone cDNA (Clontech Laboratories Inc.), and inserted between the *Bam*HI and *Eco*RI restriction sites in pcDNA4/myc-HisB. The mCherry coding sequence was PCR-amplified from pmCherry–C1 (Clontech Laboratories Inc.) and inserted between the *Hin*dIII and *Bam*HI restriction sites of the same vector. The CD63–mCherry fusion was constructed as described previously^17^. In brief, the CD63 coding sequence was amplified by PCR from a human mast cell line (HMC-1) cDNA library prepared in our laboratory and inserted between the *Hin*dIII and *Bam*HI sites in pcDNA4/myc-HisB, whereas the mCherry coding sequence was inserted between the *Bam*HI and *Eco*RI sites.

### Cell culture and transfection

RBL-2H3 rat mast cells (JCRB0023) and G361 human melanoma cells (JCRB9074) were purchased from the Japanese Collection of Research Bioresources Cell Bank (Osaka, Japan) and tested for mycoplasma contamination. HMC-1 human mast cells were provided by R. Goitsuka (Research Institute for Biomedical Science, Tokyo University of Science, Tokyo, Japan). The cells were cultured in Roswell Park Memorial Institute 1640 medium containing 10% fetal bovine serum in a humidified incubator at 37 °C with 5% CO_2_. To prevent mycobacterial contamination, cells were freshly inoculated from frozen stock for each assay. A Neon electroporation system (Invitrogen/Life Technologies) was used to deliver DNA in two 1200 V/20 ms pulses for RBL-2H3, and one 1450 V/20 ms pulse for G361. Electroporation of a 10-μL sample of ~5 × 10^4^ cells required 1.0 μg of plasmid. Transfected cells were inoculated, cultured, and imaged in a Lab-Tek chambered cover glass (8-well Nunc; Thermo Scientific, Waltham, MA, USA), which was encapsulated to prevent pH changes caused by decreasing pCO_2_.

### Live-cell imaging

Live cells abundantly expressing GFP were imaged at room temperature on a Zeiss LSM710 confocal microscope using a Plan-Apochromat 63 × / 1.40 oil correction objective and ZEN software (Zeiss, Oberkochen, Germany). The cells were visualized without changing the media to minimize stress and prevent changes in morphology. Although GFP expression in cells was not quantified, fluorescence intensity of similar magnitude was achieved under the same imaging conditions using 5 mg/mL purified GFP. Two images were acquired and averaged at pixel size 40 × 40 nm^2^ and pinhole 1.0 AU (70- and 82-μm diameters for GFP and Alexa Fluor 555, respectively). GFP was excited at 488 nm by an argon laser at 3.0% power, whereas Alexa Fluor 555 and mCherry were excited at 543 nm by a helium/neon laser at 20% power. Serial confocal images were acquired at 0.20-μm intervals. Blind deconvolution in AutoQuantX3 software (Media Cybernetics Inc., Rockville, MD, USA) was used to process the raw images in Figs 1 and 2. For rapid scan for observation of SG-SG fusion in Fig. 4, we acquired the cell using a C-Apochromat 40 × / 1.4 W objective at pixel size 88 × 88 nm^2^ and at 0.25-μm intervals.

Prior to the study, we evaluated the suitability of various fluorescent proteins for NCI in combination with dual-color imaging. Similar to GFP, ZsGreen1 (Clontech Laboratories Inc.) provided good negative contrast; however, it showed weaker expression than GFP. The yellow fluorescent protein Venus^32^ also yielded good results, but its wider fluorescence spectrum interfered with that of the red fluorescent protein mCherry used as a specific SG marker in combination with GFP-based NCI. Other fluorescent proteins with longer wavelengths also provided negative contrast, although less clearly than GFP. Similar to mCherry, DsRed monomer (Clontech Laboratories Inc.) was suitable for use in combination with GFP-based NCI; however, its fluorescence intensity was weaker. Other fluorescent proteins such as far-red fluorescent protein may be available for NCI plus dual-color imaging; however, we did not evaluate these proteins. Thus, we concluded that the combination of GFP and mCherry was convenient for NCI plus dual-color imaging.

### Preparation and imaging of microbeads

Stock solutions (100 μL) of polystyrene beads (diameter 0.5, 0.75, and 1.0 μm; Polysciences, Inc., Warrington, PA, USA) were coated for 1 h at 37 °C with 1 mL of 1% (w/v) bovine serum albumin (BSA) in phosphate-buffered saline (PBS) and were then washed three times with PBS. The beads were labeled for 1 h at room temperature with 0.5 μL of AF555 carboxylic acid (1 mg/mL in dimethyl sulfoxide, Molecular Probes, Eugene, OR, USA), washed three times with PBS, and resuspended in 100 μL of PBS. Labeled beads were immobilized on an 8-well chambered cover glass by drying at room temperature, and 200 μL of PBS containing 1 μL of bead stock was added to each well. The cover glass was blocked at room temperature with 100 μL of 1% BSA in PBS. After 30 min, the blocking agent was replaced with 100 μL of glutathione-S-transferase-GFP fusion protein (5.0 mg/mL in PBS), which was purified from overexpressing *Escherichia coli* BL21(DE3) pLysS using a glutathione sepharose column (GE Healthcare, Little Chalfont, Buckinghamshire, England). Serial confocal images of negatively stained and AF555-labeled beads were acquired at 0.1-μm intervals.

The beads were delivered by electroporation into GFP expressing RBL-2H3 cells as described, using 1.0 μL of bead stock per 10 μL of cells. The cells were allowed to adhere for 3 h before the medium was replaced. Electroporation efficiency was evaluated by microscopy the following day. The number of cells containing cytoplasmic beads was low (<10 cells/well), but higher in electroporated cells than in control cells (data not shown).

### 3D reconstruction

Prior to 3D reconstruction of SGs, all images were deconvoluted using AutoQuantX3 software (Media Cybernetics Inc.), and converted black and white by ImageJ^33^,^34^. Then, 3D images of cells were constructed from serial confocal images using Neurolucida (64-bit; MBF Bioscience, Williston, VT, USA). Contours were abstracted automatically (beads, cell body and SGs) or semi-automatically (nucleus). For fine structures, contours were defined to be the area with 90–95% fluorescence intensity when the center and periphery of a negatively stained region were set to 0% and 100%, respectively. Each contour was linked to a contour at the same position in the neighboring image, and designated as a single structure. Abstracted and grouped contours were reconstructed in 3D by lamination at the intervals used to acquire the individual images. Size and volume were calculated from reconstructions using Neurolucida Explorer (MBF Bioscience).

### Stimulation with phorbol 12-myristate 13-acetate (PMA)

To analyze SG–SG fusion, cells co-expressing GFP and NPY–mCherry in the cytoplasm were treated with 0.2 μM PMA^27^and imaged at various time points. Orthogonal and 3D images were reconstructed from serial confocal images as described above.

## Additional Information

**How to cite this article**: Tanaka, S. and Takakuwa, Y. A method for detailed analysis of the structure of mast cell secretory granules by negative contrast imaging. *Sci. Rep.* **6**, 23369; doi: 10.1038/srep23369 (2016).

## Supplementary Material

Supplementary Information
