# Supplementary material for: A method for detailed analysis of the structure of mast cell secretory granules by negative contrast imaging
Source: Sci Rep. 2016 Mar 21;6:23369. doi: 10.1038/srep23369 (PMC4800307; doi:10.1038/srep23369)

SUPPLEMENTARY INFORMATION

A method for detailed analysis of the structure of mast cell secretory granules by negative contrast imaging

Shotaro Tanaka* and Yuichi Takakuwa

Department of Biochemistry, School of Medicine, Tokyo Women's Medical University, Kawada 8-1, Shinjuku, Tokyo 162-8666, Japan

* Corresponding author. FAX: +81-3-5269-7416

E-mail address: shtanaka@research.twmu.ac.jp

SUPPLEMENTARY FIGURE LEGENDS

Supplementary Figure 1. Confocal images of a live RBL-2H3 cell expressing GFP and stained with MitoTracker (red). Insets are high-magnification views of boxed regions. Scales bars are 10 µm in the main image and 2 µm in insets.

Supplementary Figure 2. Lateral view of G361 human melanoma cells expressing GFP. Dashed lines represent cross-sections, while arrowheads mark spherical SGs. Scale bar, 10 µm.

Supplementary Figure 3. Accuracy and fidelity of NCI. Negatively stained and AF555-labeled (red) polystyrene beads (~ 1.0 µm diameter) imaged *in vitro* (a–c) and in cells (d–f). (a, d) Horizontal (top) and lateral (bottom) views of AF555-labeled beads in 5.0 mg/mL GFP (a) or in live RBL-2H3 cells expressing cytoplasmic GFP (d). Dashed lines indicate cross-sections, and the arrowhead marks the surface of the cover glass. Scale bars, 1.0 (a) and 10 µm (d). (b, e, c, f) Theoretical (dashed line) and observed bead diameter (b, e) and volume (c, f). Error bars are standard deviations from 12 (b, c) and 5 (e, f) measurements. Insets are representative 3D images of beads. Scale bars, 1.0

µm.

Supplementary Figure 1

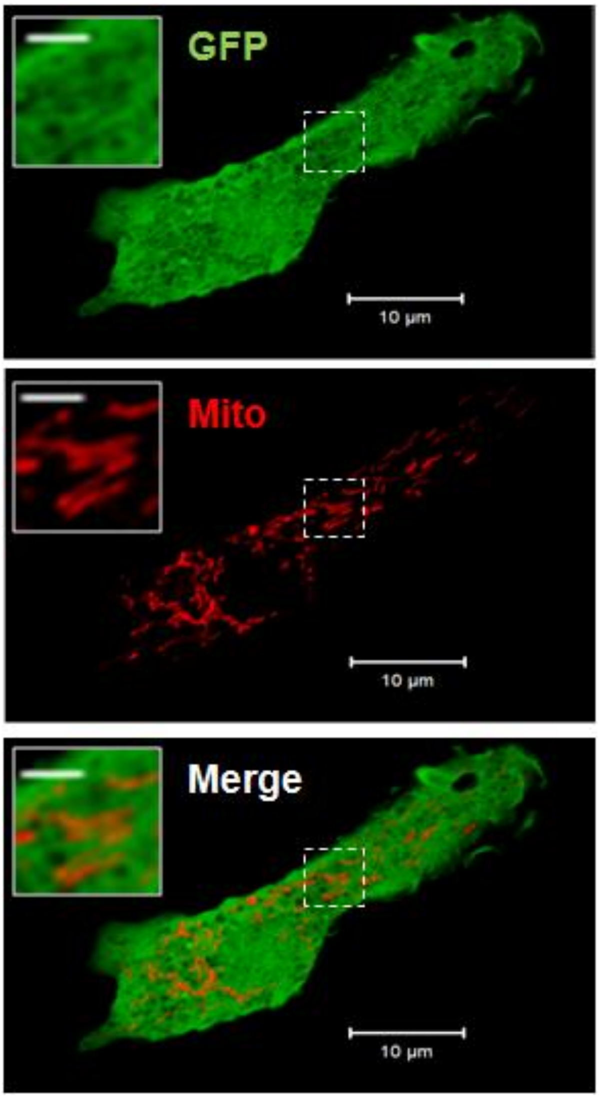

Supplementary Figure 2

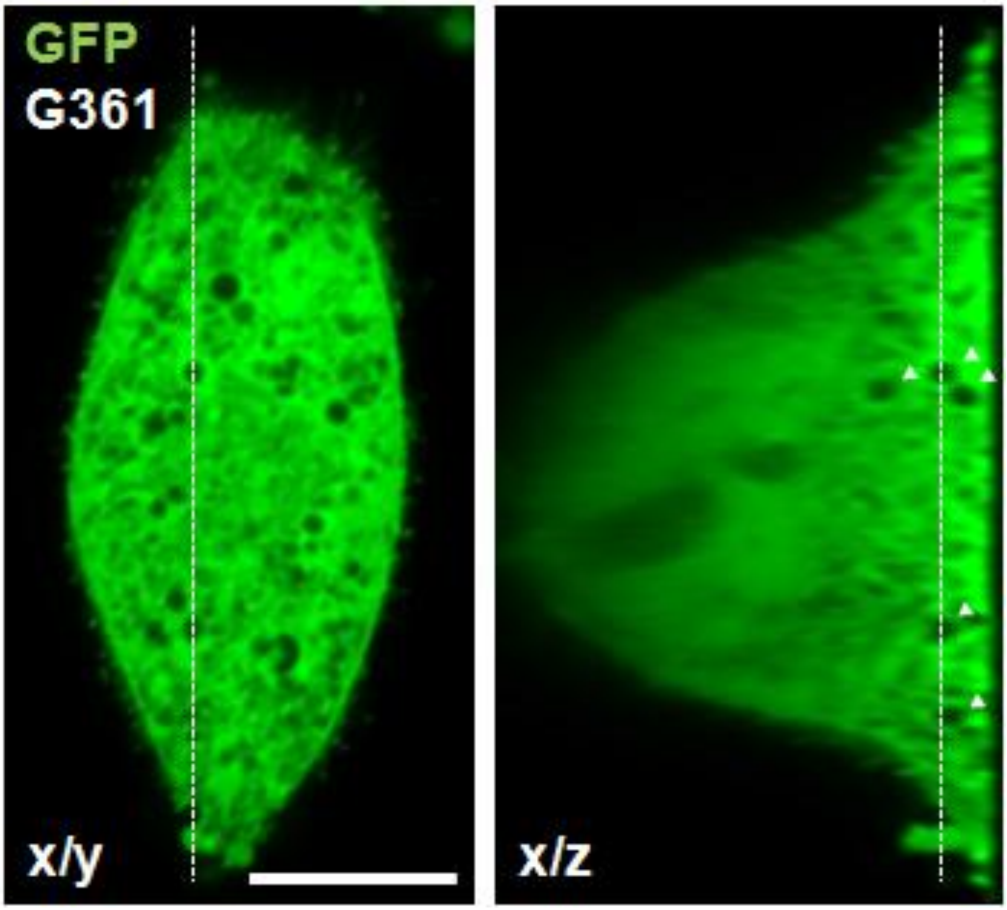

Supplementary Figure 3

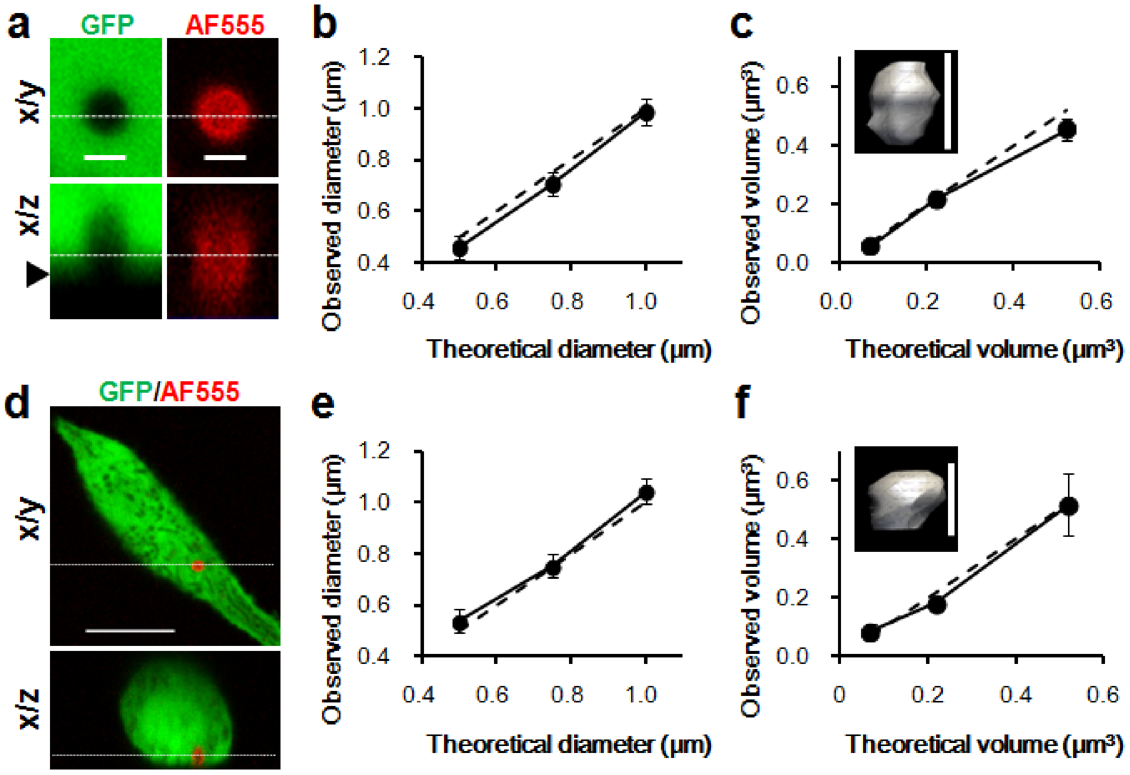

Supplement: Supplementary Information [file srep23369-s1.pdf]
